# Developing Simulated and Virtual Patients in Psychological Assessment – Method, Insights and Recommendations

**DOI:** 10.5334/pme.493

**Published:** 2023-10-27

**Authors:** Bartosz Zalewski, Mateusz Guziak, Maciej Walkiewicz

**Affiliations:** 1 Department of Psychology of Individual Differences, Diagnosis and Psychometric Research, SWPS University of Social Sciences and Humanities in Warsaw, Poland; 2 Department of Family Medicine, Faculty of Medicine, Medical University of Gdańsk, Poland; 3 Department of Psychology, Medical University of Gdansk, Poland

## Abstract

The phenomena of the simulated (SP) and virtual patient (VP) is widely described in the literature. Although it is difficult to find any practical information on developing these methods for teaching psychological assessment. Having conducted a long-term research project regarding this topic, we report the experience gained and retrospectively identify many mistakes. In this article, we present a summary of creating and using both SP and VP methods in clinical psychology and propose some insights and tips for their development, based on our experiences. While the project concerned clinical psychology, we believe the reflections might be applicable to a wider group of educational situations in which students develop competencies to carry out a diagnostic process with a real patient.

## Background & Need for Innovation

The teaching of psychological assessment is usually based on the participation of real patients [1]. However, this method has many limitations, such as difficulties with the availability of patients, or is burdened with many ethical controversies. Students struggle with a sense of great responsibility for patient welfare, which increases their level of tension and leads to committing mistakes [2,3,4]. Both methods described in the paper – simulated (SP) and virtual patient (VP) – are designed to eliminate the weaknesses associated with real patients. They also prove to be easily adaptable to online teaching.

Our simulated patient method refers to Simulated Standardized Patient used in medical training. However, it is rarely used in teaching psychological competences. Students conduct a diagnostic interview with a person specially trained to present symptoms associated with specific medical conditions. We began our work on the SP after having identified existing practices for the use of live simulation in medical and psychological education [5,6,7]. Virtual Patient on the other hand, is defined as Interactive Patient Scenarios for health, treatment, education and measurement purposes [8]. VP technology has become one of the most popular types of problem-based learning in medical education [9,10]. Its main focus is on simulating clinical steps including patient history, physical examination; laboratory tests, diagnosis, treatment [11] and communication skills that form the basis of clinical practice [12].

Both methods reflect two learning conditions: direct contact with a living person (SP) and with computer-mediated (VP). SP requires significant involvement and is potentially burdensome for the learners, since they had to perform a complex diagnostic activity that involves simultaneously: building contact, collecting diagnostic data, processing information, ascribing the clinical symptoms, controlling emotional reactions, bridging the results of cognitive biases, formulating diagnostic interventions and more [1,4]. VP – integrates a lower cognitive-emotional burden, since the student performs the diagnostic tasks without direct contact, and, at the same time, can be regularly provided with feedback about his/her steps [9].

Although the phenomena of the simulated (SP) and virtual patient (VP) is widely described in the literature, it was difficult to find any practical information on developing these methods for teaching psychological assessment.

## Goal of Innovation

In this article, we present a summary of creating and using both SP and VP methods in clinical psychology and propose some insights and tips for their development, based on our experiences.

## Steps Taken for Development and Implementation of Innovation

The background for this paper is a wider research project ‘Diagnostic competence in the area psychological clinical diagnosis – development conditions’, supported by the National Science Centre, Poland. The project goal was to describe how to effectively teach the competencies of conducting clinical diagnosis in psychology. The effectiveness of learning was measured with SP and VP. At the same time, the study sought to find the psychological characteristics of students that influence their learning efficiency with each of the methods. Cognitive and emotional features were examined. We verified several different hypotheses concerning various competencies or skills, such as conducting an interview or formulating a diagnosis. Two following manuscripts include the methodology of the whole research project with appropriate tools and examples [13,14]. Additionally, in Appendix 1 we present its scheme (see Appendix 1).

### Simulated patient development

#### SP prototype

It was created by a team of clinical psychologists/scientists from the Academic Psychotherapy Centre at SWPS University of Social Sciences and Humanities, Poland. Eight patient profiles were prepared for the simulations (see Appendix 2), inspired by actual cases from the Centre and conceptualized in one of the mental health theories. It resulted in the construction of 4 types of profiles ranging patients from healthy to one with severe mental disorders [15]. The type of disorder was described by indicating: the dominant symptoms, the level of reactance, readiness for change and the style of coping.

#### SP simulants

The next step was to build reliable and consistent simulations. A team of 12 SP simulants met once a week for about 2.5 hours for one semester to discuss the methods of role-playing. Then the simulations were exercised. The emotional consequences and burden of playing them for the simulants were discussed in a group and considered in the further development of simulations. The role-playing was prepared for a full 50-minute diagnostic session to create the most realistic conditions for psychological consultation. Candidates for the SP were pretested with questionnaires covering both their psychopathology and psychological well-being. Each of the qualified SPs voluntarily chose one profile to play.

### Virtual patient development

Our VP method consisted of short recordings presenting statements of patients. Role scripts were created on paper, without prior recordings. The same patient profiles were used to build the VP and SP (see Appendix 2). Then, based on scripts, scenes/elements of the interview were recorded on video. Finally, the scenes were arranged in accordance with the decision tree in the role scenarios. When building the script, each diagnostic intervention needed to be properly described. Due to this, the program could assign each question and give feedback. This description included the type of intervention, evaluation, and an indication of what to do next. The student received feedback after each choice: proper, and improper intervention. ‘Proper’ meant well-constructed diagnostic questions and contact-building statements applied at the right time in the interview. ‘Improper’ contained various basic errors, such as advising, suggesting or assessing instead of asking. In the end, VP covered a 20 minutes diagnostic interview (films + diagnostic reactions).

The participant watched the initial VP statement and needed to select one of two possible reactions. Depending on the selected reaction, the program played a video connected with this choice, containing the patient’s next statement. Then, subsequently, the participant chose the next reaction and watched the next video. The procedure was continued for nine rounds. The training part was realized through feedback, generated by the program immediately after the selected reaction. Also, right after the training was over, the participant received standardized feedback on the screen. The participant’s task was to interview the VP according to the rules s/he learned in the training course at the beginning of the research program. The computer program saved all the participants’ choices, which were automatically evaluated.

## Evaluation of Innovation

### Simulated patient evaluation

During preparations, we discovered that the simulants struggled to remember the details of their roles. Parallel, participants asked about areas that were beyond the simulants’ profile. Later on, it turned out that these were not a problem when SPs could improvise based on profile’s psychopathology. On the other hand, it revealed an issue related to the limits of improvisation, as the simulation was not strictly standardized, so it differs from Standardized Patient method. We wondered how many details the simulants should invent themselves. It was decided that simulants would build a more developed character and expand it with a variety of thoughts, feelings and behaviours, based on the main psychopathological mechanism they perform. This was an authorial part of the work for each SP based on the profile received. There was the question of whether the simulants were to use her/his feelings and reactions to the student’s behaviour during the interview, or were only to imagine how the subject of the profile would feel and react. Eventually, it was considered that simulants should use their natural reactions, even if they were not ideally consistent with the profile. This solution allowed a sense of building up real contact with the live-acting simulant. Later on, we noticed that thanks to this decision, elements of important phenomena: transference and counter-transference appeared in those conversations.

We also noticed a specific pattern: the simulants playing less disturbed patients tended to increase the symptoms in their play. As a result, they played a person with a deeper disorder than planned. Paradoxically, the easiest character to play was the deeply disturbed one, while at the same time, it was the most emotionally charged. In contrast, it was most difficult to play a healthy person, although it was the least emotionally exhausting. Probably the symptoms of disorders were more clear, more obvious and easy to play. It could also be that they might have tried to help participants by play intensive and clear symptoms.

The literature shows an absence of deeper or long-term consequences of playing the role of patients with serious mental disorders or traumatic experiences. Only inconveniences occurring immediately after playing the role were described [6,16,17,18]. Our research has indicated that repeated playing of such a role may have a negative impact on the well-being of simulants [4]. Initially, students were hired to play SPs, but they were further replaced with clinical psychologists. The difficulty with the psychology students as simulants was that when playing the role, they tended to explain the patient’s problem rather than play it out. Students were generally more reflective than real patients, because of their knowledge of psychology studies and they may empathizing with participants – who also were psychology students. Another problem with the students as simulants were the limited imagination of the various symptoms of the disorder and the intensity of these symptoms turned out to be student-simulants’ weaknesses. Following these observations, in the second research, we changed people who played simulations – in the pilot study simulants were performed by psychology students, but in the main research we decided to hire clinicians to simulate patients.

Generally clinicians are more effective in training: they needed less time to prepare for the role – students needed about 10 hours while clinicians 3–4. For clinicians, it was much easier to imagine the symptoms of mental disorders. Eventually, it turned out that for a good role calibration it was necessary to have at least two plays, and after 4–5 plays, a person became fluent in the simulation. Clinicians were also more spontaneous as simulants than students. It was important because the more spontaneous the simulants were, the more effective the simulations occurred. What is more, it was helpful for clinical psychologists to use moments of silence and reverie during the role-play. When they did not respond immediately from participants performing the interview with them, they started to get tangled up, silent, withdrawn from contact and gave signals of resistance, which turned out to be very realistic and reflected the contact with real patients. This allows the phenomenon of transference to occur in the contact between the participant and the simulant. Real patients often do not know how to answer the diagnostic questions and because of strong emotions experienced during the consultation, it is difficult for them to focus on formulating coherent answers – and it was well played by simulants.

We decided not to hire professional actors, due to the difficulties in leaving the role, especially when simulating patients with deeper disorders [17,19]. We believe that the advantage of clinical psychologists and psychotherapists over actors in simulations of mental health disorders may be based on specific professional experience. Clinicians are trained and practiced in their daily work to deal with difficult emotions evoked by the patients (like vicarious trauma/secondary traumatic stress). This is due to the specificity of postgraduate training, e.g. self-psychotherapy and supervision of therapeutic work, but not only. The work of clinical psychologists and psychotherapists is largely team-based, and is related to constant relations with the clinical team, peer supervision, conducting family therapy together with a co-therapist, etc.. In clinical work, there is a large amount of interaction with other clinicians, which regulate emotions through the social networks of psychotherapists. As a result, they seem to be less emotionally burdened by playing the role of simulated patients presenting psychological problems. We believe it worked in our research – in the case of deterioration in psychological well-being, they could withdraw from further role-playing at any time and use their training to regulate their emotions. Overall in the two parts of the research (pilot study and main study), 294 simulations were used. No negative consequences of simulations for the simulators were observed.

Simulants revealed that some experiences during the interviews were frustrating and sometimes unpleasant or annoying. It was especially difficult when participants prolonged the interview or committed many mistakes, like judging, interpreting or giving oversimplified advice. Also, when the participants were concentrated on building contact, they rarely asked diagnostic questions. In those situations, although the atmosphere of the consultation was conducive to building diagnostic alliance, the participants did not obtain the necessary information to formulate the diagnosis. In other situations, SPs had the impression that the patient they were playing evoked the diagnostician’s/participant’s anger – they often finished before the end of the interview. Unfortunately, in our project, some participants seemed to present significant psychological difficulties (an estimated 5–10% of the participants). The contact with them was very burdensome for the simulants, as it would be with real patient as we believe.

The length of the session also turned out to be important. We concluded that a 50-minute interview proved to be a mistake. In the literature, we did not find any such lengthy simulations. In our study, it was usually the first full-length clinical interview for participants to conduct and it appeared to be both cognitively and emotionally exhausting. We noticed that the students can collect diagnostic information sufficiently for about 20–25 minutes. Then they either finished the interview or their effectiveness deteriorated significantly and they start to committing many mistakes e.g. giving advises about the eating habits, or concerning spiritual and religious practices.

Our next problem with SP was dropouts. Participants often did not show up for the second or third meeting with the SP (they have from 2 to 4 interviews with the SP, depending on the training mode they were assigned to). We think that this was the consequence of the participants’ psychological overload after the first meeting.

### Virtual patient evaluation

We have checked several ways to construct the Virtual Patient. Our first attempt was to build VP, using the same patient profiles as were used to build the SP. It was planned to build the VP just by using recorded interviews with the SP, which would be cut and edited into one continues VP interview. It appeared to be impossible, as it was difficult to create a coherent storyline consisting of recordings from several live interviews. In the end, we developed a script consisting of short scenes arranged in the play order according to the decision tree. Our conclusion is that the decision tree cannot be too extensive. Originally, it was planned that after each patient’s statement, there were three interventions to choose from: proper, improper or mixed intervention Yet, finally, dichotomic paths were used: proper and improper. Eventually, 512 possible paths were created. Building such a decision tree was quite a simple task, but very time-consuming due to the number of paths. It took the team of three researchers about 250 hours to create a whole decision tree. With three interventions the total number of paths would be 19,683, which is impossible to create due to enormous amount of work needed.

The significant advantage of the VP over SP was the fact that it was not necessary to train simulants for role-playing. In the VP method, patients’ statements are not continuous but are made in small fragments, so there is no emotional burden for the simulants. Patients do not need to be played by professional actors, students or clinicians.

VP is a safe method for participants since it gives extra time to choose the right intervention. In general, clinical students report that one of the most difficult things in learning clinical diagnosis is the need for an immediate response [20]. In this method, they heard and saw patients’ statements, which were often highly emotional, and they had extra time for emotional regulation and evaluation of the information obtained. What is more, they did not have to worry about the iatrogenesis of the patient, which is an advantage over using the real patient’s method [2,3,4].

Our experience also shows that while creating the VP method, the team should consist of IT and UI/UX specialists from the very beginning. Of course, we are aware that this may be prohibitive, for example, due to costs and availability. In addition, due to the high amount of resources involved in creating such methods, it makes sense to build them only if they are intended to be used extensively.

## Critical Reflection of Innovation

From our perspective, each method described above has specific advantages and disadvantages. The advantage of the SP and VP over real patients we believe is that participants know that there is no real person on the other side. This may be alleviated by the fact that the student sees the SP or VP as a person sitting in the psychologist’s office and playing real disorder symptoms and is built based on a real case.

It should be noted that the effort required to develop and apply the SP and VP in our experiences appeared to be distributed differently. The VP method takes a lot of effort and time to prepare but is then simple to apply in a variety of teaching settings and multiple uses, while the SP is fairly simple to build – prepare and learn roles – but the service is demanding. The use of the SP requires arranging several people for each meeting: a simulant, an observer and the person being examined. The application of the VP requires only sending a link with the VP to the student or sharing the computer with the VP installed.

In the conducted project, the cost of implementation of both programs was similar. In the VP, it was primarily expensive to prepare the decision tree. It requires the work and time of several specialists to prepare material for recordings and to build a suitable virtual environment. Later on, using the VP is almost cost-free, apart from maintaining the platform and technical service. In the SP, the costs of research and development were lower (covers the cost of 10 hours of training for each student simulant and 3–4 hours for the clinician simulant), while the costs of using the SP were high: with each session requiring several hours of work by at least two specialists. In Table 1 we present some recommendations for creating SP and VP methods.

**Table 1:** Recommendations for creating SP and VP method.


1.	The more spontaneous the SP was, the more effective were the simulations. SP should be dynamically reactive: if participants perform a proper interview, they should be rewarded with an extensive response.

2.	In the short term, it seems easier to play a more distorted profile of the patient, which is probably easy to adopt, even though it is more emotionally exhausting for SPs. The limit here is how long the role is played, as long-term exposure may correlate negatively with the simulant’s well-being. In general, SPs should use their natural reactions, without worrying too much whether the reaction is consistent with the profile.

3.	Participants who are students can perform the interview effectively for no longer than 20–25 minutes.

4.	The involvement of IT and UI/UX specialists from the early stages of creating the VP would be extremely helpful.

5.	Due to the relatively high amount of labour involved, creating such methods for extensive, long-term use seems to be optimal.


## Additional Files

The additional files for this article can be found as follows:

10.5334/pme.493.s1 Appendix 1. The scheme of the research project.

10.5334/pme.493.s2 Appendix 2. Examples of SP and VP profiles.
