# Supplementary figures and images for: Developing Simulated and Virtual Patients in Psychological Assessment – Method, Insights and Recommendations
Source: Perspect Med Educ. 2023 Oct 27;12(1):455–61. doi: 10.5334/pme.493 (PMC10607628; doi:10.5334/pme.493)

## APPENDIX 1

### The scheme of the research project

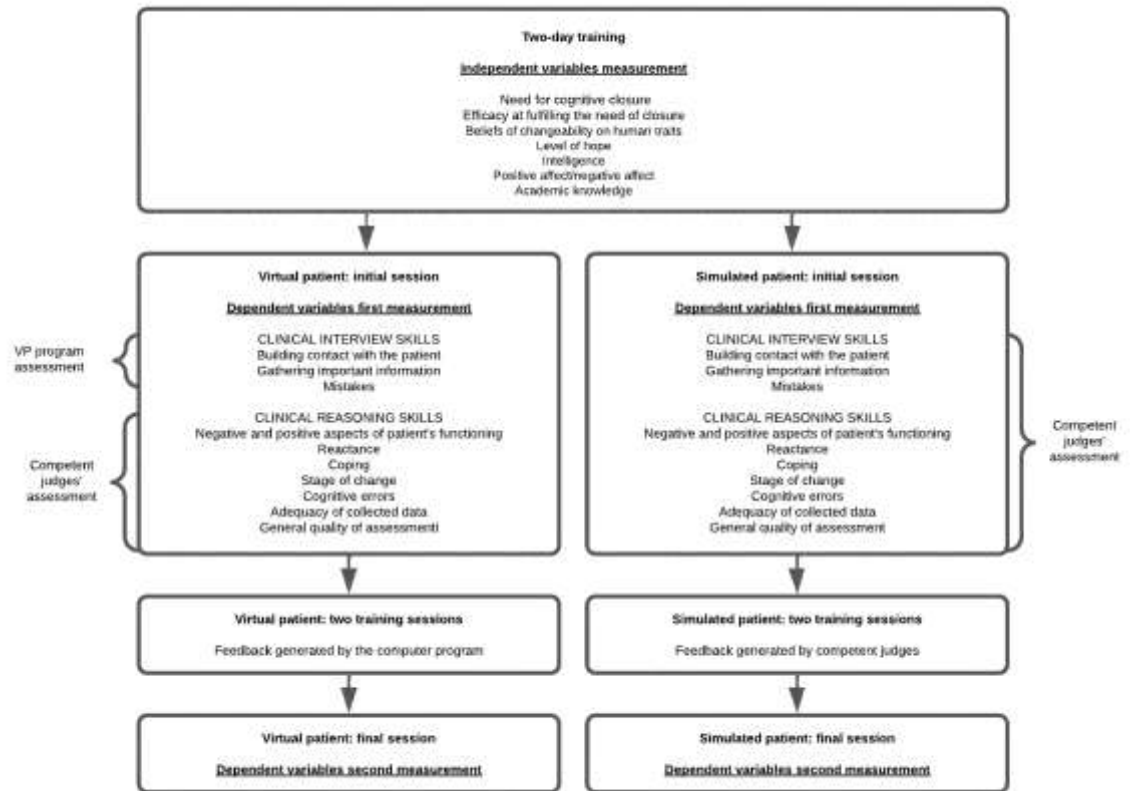

Supplement: Appendix 1. — The scheme of the research project. [file pme-12-1-493-s1.pdf]
