# Supplementary material for: Developing Simulated and Virtual Patients in Psychological Assessment – Method, Insights and Recommendations
Source: Perspect Med Educ. 2023 Oct 27;12(1):455–61. doi: 10.5334/pme.493 (PMC10607628; doi:10.5334/pme.493)
Supplement: Appendix 2. — Examples of SP and VP profiles. [file pme-12-1-493-s2.pdf]

APPENDIX 2

Examples SP and VP profiles

In Appendix 1 we provide examples of the individual patients played with the full description of the problems reported by the patient, the duration and severity of symptoms, a short history of the patient's personal and professional life, and the environment from which s/he came from, including a history of romantic relationships.

Profile 1, Miss X, 33

The patient visits the therapy centre because she would like to take better care of herself. She works as a choreographer. She is highly valued for her competence, but still only holds the position of assistant, not the main production choreographer. She dreams of being noticed and discovered but thinks that there are many jealous, unkind women in her environment, who compete unfairly. The patient would not like to compete the way they do – she values good relations, does not want to stand out at the expense of others. At the same time, she highly values her professional competence. She would like to be able to effectively strive for a position that would reflect her competencies, at the same time she would like to be able to do it her own way – without entering into the unhealthy competition.

Currently, she is in a relationship with her partner – D., who is a wealthy businessman. They live in a house near the capital city of [COUNTRY] with a beautiful garden, a fountain, and their own conservatory. However, being there, away from people, and the city She declares that she works at her best among people. She organises her social life for herself and her partner. She describes her partner as cool, slightly withdrawn, showing feelings in a very reserved way.

The patient is emotionally in a very difficult situation – as she explained, tired of the relationship with a cool partner, for the first time in her life she got involved in an affair with her colleague M. He is brilliant – very talented, sociable, affectionate. The patient feels that he is her 'soulmate' – a man with a passion for life, with interests that are close to her; he is like a male version of herself. The patient feels that she has been waiting for someone like that all her life. At the same time, she doesn't feel strong enough to end her relationship with her partner. She told him about the affair – she wants to be honest in her relationship. D. was angry, but he decided to forgive her and strive to work at their relationship. He says that the patient is the most important person in his life.

The patient is trapped – she loves M. but doesn't want to be disloyal to D. Patient's mother advises her to end the affair, commenting that the relationship with M. is yet another relationship in the patient's life and it may be that after 2 or 3 years she will become bored with her relationship with M. She is already 33 years old and it would be good to stay in a stable relationship. The patient acknowledges her mother is partly right, but she doesn't want to duplicate her mother's own story – her mother is in an emotionally distanced relationship with her father. The father is highly placed in a largely state-owned company – he earns a lot, he is often not at home. He always emphasised that patient must not bring shame on him. The patient feels very sorry for her mother about this relationship – she understands that her mother had to look for warmth in relationships outside of marriage. However, she would like to be in a relationship with someone whom she loves and who shares her passions and energy for life and knows how to show her commitment.

<u>Example of a situation (work)</u>

Recently, the patient was employed in the production of a short film. She was very pleased with the results of her work, but the director did not praise her. The production budget was large – it was clear nothing was saved. The patient was employed by the head of the choreography. After implementation, the boss delayed the transfer of payment for a long time – it was frustrating. The patient later learned that she earned a fraction of what the boss did, although she did almost nothing – all the best ideas were the patient's ideas. This situation aroused to anger, indignation and powerlessness – the patient would not dare to apply for an independent position, going against the boss who had previously hired her.

<u>Example of a situation (relations)</u>

The patient came to [CITY] last week because of the promotion of the film on which she collaborated. Her partner, D. forbade her to participate in the informal celebration that took place after the official event. Her friends were very surprised that she was leaving and they started calling her. In the end, she gave in and joined them. M. - with which she had an affair - was also there. They went for a walk together, talked for a long time – it was amazing as if they had known each other for many years. Later, however, the patient decided that she was behaving badly, she barely said goodbye and went to the friend she was supposed to be sleeping over with. Her friend was

surprised that she came without M. and suggested that they go over to M.'s together. The patient arrived and then after several minutes, the friend said goodbye. The patient finally spent the night with M. – as she states, it was incredibly close and significant, but she felt she was being unfair towards D.

Expectations for the therapy:

- get a job as the first choreographer, be able to take care of her needs in her personal life, be good at making choices in a way that does not hurt others.

Profile 2, Miss Y, 26

She is in a relationship, but she is not able to start having sexual life with her partner. She is also very tired in her work – she works in a clothing store in a large shopping centre.

She is highly valued at work, but she works over 12 hours a day, she doesn't have free weekends, she often works at unfavourable hours, and she is stating that the boss is harassing her. She is afraid of getting angry with the boss and does not refuse him. She is afraid that she will not find a better job. She doesn't feel confident enough to send a CV to another place.

She studies journalism – she is doing great, she has a scholarship and a lot of support from the faculty authorities (lecturers appreciate her commitment and competence). She has a large group of friends and two close ones. Together they go on vacation to the lake district in [COUNTRY]. They like to read, talk and spend time together.

Now she lives with her boyfriend. Decorating their new place gave them a lot of joy. They live with the patient's boyfriend's father, who often goes away and does not interfere in their affairs – she and her partner often have the whole apartment to themselves. Recently they adopted a cat who they called Harold. The client values contact with the animal very much.

Currently, she is afraid that her lack of ability to have intercourse will harm their relationship. She is afraid that her boyfriend will leave her, although he declares that this is not true. However, she thinks he can't stand
it any longer. The patient is afraid that a relationship without an element of intercourse is so unsatisfactory for a man that she expects that if doesn't feel able to have sex with her boyfriend soon, he will start cheating on her or will leave her. The partner finances her psychologist consultation and wants to finance her therapy because she doesn't earn very much. The patient would like to be able to have the courage to change jobs. She would like to earn more so as not to be a burden to her boyfriend – she wants him to be able to afford nice clothes and enjoy himself.

When she thinks about changing jobs, she is afraid that people reading her CV will laugh at her lack of experience. She is afraid that even if she finds another job, she will not be better in it than in her current job, that she will be a victim of harassment again. She feels an irrational fear of men who remind her of a man who has molested her in the past. It was a father's friend; the patient does not want to talk about this anymore. She is afraid that she might meet someone similar to this man at an interview or a new job. She knows that it would paralyse her – she wouldn't be able to speak and she couldn't work.

She would like to undergo therapy focused on dealing with the fear of intercourse and the fear of looking for a new job. She emphasizes that she doesn't want to work with a sexologist because she thinks her problems are emotional, not physical.

An example of a situation in which the patient was afraid of her boyfriend leaving her

Recently, when she was with her partner in a shopping centre he noticed a dress in the shop and asked her to try it on. The patient put on the dress and could see that she looked attractive in it. The boyfriend said that she looked great. However, she thought that she was now attracted to him and she felt horror – a tightness in the stomach and tension in the arms and neck. She thought that he would like to have sex with her, and she was very afraid
of it, she really does not want to have sex. She thought her reaction was abnormal – she taught that women like to please their partners. She felt guilty, strange. She thought her boyfriend wouldn't be able to tolerate it in the long run. She told him that she did not like this dress and immediately hung it up. The whole evening was tense and sad, she wanted to cry. At the same time, she tried to hide it from her boyfriend.

An example of a situation in which the patient was afraid to look for a new job

Yesterday, the patient looked at job offers in the newspaper. She began to check if she had the skills required for several positions that seemed attractive to her. First, she decided that she actually had these skills, and then she imagined that she would be asked about them during the interview (e.g. the interview would be in English). She imagined how she was losing the thread in the interview, and the interviewer comments on her lack of skills and accuses her of lying – she had written that she knew English. The patient feels fear paralysing her – she pushes the newspaper away and decides not to think about it anymore.
